# Investigation into the prevalence of a novel dendritic-like cell subset *in vivo*

**DOI:** 10.1111/jcmm.12174

**Published:** 2013-11-19

**Authors:** Kristin Lisa Griffiths, Jonathan Kah Huat Tan, Helen Christine O’Neill

**Affiliations:** Research School of Biology, The Australian National UniversityActon, ACT, Australia

**Keywords:** Dendritic cells, cross-presentation, spleen, bone marrow

## Abstract

A novel dendritic-like cell subset termed L-DC was recently identified in murine spleen based on marker expression of a homogeneous cell population derived from long-term culture of neonatal spleen. The function of L-DC is distinct from other splenic dendritic and myeloid cell subsets because of their high endocytic capacity and their ability to cross-present antigen to CD8^+^ T cells. This paper shows the subset to be unique to spleen and blood, with a similar, but possibly functionally distinct subset also present in bone marrow. The prevalence of the subset is low; ∼6% of all dendritic and myeloid cells in the spleen and ∼5% in blood. However, they are a distinct cell type on the basis of marker expression, and endocytic and T-cell stimulatory capacity. Attempts to identify an enriched population of these cells in mutant mouse strains with reported increases in myelopoiesis showed either a lack of L-DC or an altered phenotype reflective of the phenotype of the mouse strain.

## Introduction

The importance of dendritic cells (DC) in the immune system as the mediators between innate and adaptive immunity has only recently become apparent. As a result, this field of study is relatively new and DC are emerging to be a complex cell type with extensive variability in phenotype and function. Murine DC can largely be divided into three types: migratory DC, conventional (c)DC and plasmacytoid (p)DC. Migratory DC, such as Langerhans cells, develop in the periphery and migrate to lymph nodes following encounter with antigen to activate T cells 1–2. Conventional DC are blood-derived, reside in the spleen, and can be further divided into subsets based on marker expression. Functional definition of the cDC subsets, namely CD8α^+^ cDC (CD11c^+^CD11b^−^MHC-II^+^) and CD8α^−^ cDC (CD11c^+^CD11b^+^MHC-II^+^), has proven difficult. CD8α^+^ cDC are generally thought to be cross-presenting cells inducing a CD8^+^ T-cell response 3 with an ability to induce Th1 CD4^+^ T cells through production of the Th1-inducing cytokine interleukin (IL)-12, and can also produce the type I interferons IFN-α and IFN-β 4,5. CD8α^−^ cDC are classified as Th2-inducing cells 4–5; however, they have also been reported to produce IL-12 as well as IFN-γ 6–7, and have cross-presenting capacity in some model infections 8. Plasmacytoid DC are an IFN-α-producing subset involved in virus clearance at the level of the innate immune response, later maturing to become DC capable of inducing adaptive immune responses 9.

This laboratory has identified a dendritic-like subset that develops as a homogeneous population of cells *in vitro* following culture of whole spleen from neonatal mice 10. These long-term cultures maintain production of cells over a period of years, through development of large mature cells along with progenitors and precursors, which are maintained in the cultures. Long-term culture DC (L-DC) have a characteristic phenotype as CD11c^lo^CD11b^hi^CD8α^−^MHC-II^−^ cells, which are very large in terms of forward scatter (FSC) when examined using flow cytometry 10. On the basis of the distinct marker expression and size of L-DC, we recently reported an *in vivo* equivalent dendritic-like cell with high endocytic and cross-presenting capacity *in vitro* 11. The L-DC marker expression relative to other DC subsets as reported in Tan *et al*. 11 is summarized in Table 1. Here, we characterize L-DC in terms of prevalence in relation to other known DC subsets in spleen and other tissues. Given their high CD11b expression, L-DC are thought to be of myeloid origin. To determine the effect of mutations affecting myelopoiesis on the relative prevalence of cDC, pDC and L-DC, we investigate their tissue distribution and lineage relationships and examine the prevalence of these cells in normal mouse lymphoid tissues and in two mutant strains of mice where myelopoiesis has been affected.

**Table 1:** Marker expression on L-DC compared with *in vivo* splenic DC subsets

	L-DC	CD8α^+^ cDC	CD8α^−^ cDC	pDC	Monocytes
CD11c	Lo	Hi	Hi	Lo	−
CD11b	Hi	−	Lo	−	+
CD8α	−	+	−	+	−
MHC-II	−	+	+	Lo	−
MHC-I	+	+	+	+	−/Lo

## Materials and methods

### Animals

Specific pathogen-free female C57BL/6J (CD45.2^+^), C57BL/6J.SJL (CD45.1^+^) and CBA/H mice were obtained from the John Curtin School of Medical Research [JCSMR: Australian National University (ANU), Canberra] and used at 6 weeks or 8 days of age. Organs from s*anroque* mice 12 were donated by Dr. Carola Vinuesa (JCSMR). Rag KO (*Rag*^*−/−*^) mice were obtained from the Australian Phenomics Facility (APF: ANU). Ovalbumin-specific MHC-I- and MHC-II-restricted TCR-transgenic (tg) mice [OT-I (anti-K^b^) and OT-II (anti-IA^b^), respectively] were obtained from the APF. Mice were housed and handled according to protocols approved by the Animal Experimentation Ethics Committee at the ANU and killed by cervical dislocation.

### Tissue culture

DMEM was supplemented with 10% foetal calf serum (JRH Biosciences, Lenexa, KS, USA), 10 mM Hepes (JRH Biosciences), 2 mM L-glutamine (JRH Biosciences), 100 U/ml penicillin (JCSMR), 100 μg/ml streptomycin (JCSMR) and 5 × 10^−5^M 2-mercaptoethanol (BDH Ltd., Poole, United Kingdom). This is referred to as supplemented DMEM (sDMEM). Cells were maintained in 5% CO_2_ in air and 97% humidity at 37°C. For estimation of cell number and viability, cells were stained with trypan blue (0.4%/saline; Gibco BRL, Grand Island, NY, USA).

### Preparation of lymphoid cells *ex vivo*

For preparation of single cell suspensions, bone marrow (BM) was isolated from the femur of a C57BL/6J mouse. Marrow was flushed from the bone cavity by injection of DMEM by using a syringe equipped with a 26-G needle. Cells were dissociated by pipetting and resuspended in sDMEM. After sedimentation, red blood cells (RBC) were removed by lysis in RBC lysis buffer (140 mM NH_4_Cl, 17 mM Tris base in deionised water). Cells were then washed twice in sDMEM and counted. For preparation of thymocytes and cells from the mesenteric lymph node (MLN), tissues were dissociated by pressing through a fine mesh sieve. For isolation of splenocytes, dissected spleen was pressed through a fine mesh sieve. Cells were resuspended in sDMEM, RBC lysed and cells washed as described for BM. Cells were counted after the final wash.

For T- and B-cell depletion, splenocytes were incubated with 0.25 μg/ml biotinylated anti-CD19 antibody (eBiosciences, San Diego, CA, USA)/10^8^ cells (B cells), and 0.2 μg/ml biotinylated anti-Thy1.2 antibody (eBiosciences)/10^8^ cells (T cells) in 1 ml/10^8^ cells for 10 min. on ice in labelling buffer (0.5%/2 mM EDTA/PBS). Cells were washed twice with labelling buffer and anti-biotin microbeads (Miltenyi Biotec, Auburn, CA, USA) were added at 13 μl beads/10^8^ cells in 1 ml/10^8^ cells and incubated for 25 min. on ice. Cells were washed once in labelling buffer and resuspended in 500 μl of the same buffer. The LS depletion column (Miltenyi Biotec) was prepared by placing it in a magnet (SuperMacs; Miltenyi Biotec) and washing once with 3 ml labelling buffer. The cell suspension was then run through the column and collected in a new tube, followed by three washes with 3 ml labelling buffer. Cells were pelleted and resuspended in sDMEM for counting and antibody staining.

### Antibody staining of cells and analysis and sorting by using flow cytometry

Fluorochrome-conjugated antibodies were used for cell surface staining to identify subsets of interest. They were purchased as affinity-purified preparations from either eBiosciences, BD Pharmingen (San Jose, CA, USA) or Molecular Probes (Eugene, OR, USA), conjugated with either fluorochromes or biotin. Anti-CD16/32 (clone: 93), anti-CD11c-allophycocyanin (APC) (clone: N418), anti-CD11b-phycoerythrin (PE)-Cy7 (clone: M1/70), anti-CD19-biotin (clone: eBiolD3), anti-Thy1.2-biotin (clone: 30-H12), anti-CD45RA-biotin (clone: RA3-6B2) and anti-CD45RA-PE (B220, clone: RA3-6B2) were obtained from eBiosciences. Anti-CD8α-PE (clone: 53-6.7), anti-IA^b^-fluoresceinisothiocyanate (FITC) (clone: AF6-120.1) and anti-IA^b^-biotin (clone: 25-9-17) were obtained from BD Pharmingen. Streptavidin-APC-AlexaFluor-750 was obtained from Molecular Probes. All antibodies were titrated prior to use to determine the concentration giving minimum saturation binding.

For staining, ∼5 × 10^5^ cells were sedimented into the wells of a flexible 96-well polystyrene microtitre plate (Corning, Tewksbury, MA, USA). Cells were resuspended in anti-CD16/32 (FcBlock) prior to staining in a primary antibody cocktail. Where necessary, secondary reagents were added before resuspension in DMEM/1%FCS/0.1%NaN_3_ before transfer to cluster tubes (Corning) for analysis by using a flow cytometer (Becton Dickinson LSRII, San Jose, CA, USA). Prior to analysis, propidium iodide (PI: 100 μg/ml) was added for dead cell discrimination. Background binding was determined by using isotype control antibodies corresponding to each fluorescence-conjugated antibody used, and gates were set by using this information as well as internal positive and negative cell subsets to identify specific staining. Analysis was performed using BD FACSDiva software (Becton Dickson) and Flow Jo software (Tristar, Phoenix, AZ, USA). For fluorescence-activated cell sorting, cells were processed as above before being sorted at the sorting facility at the JCSMR.

### Endocytic capacity of cells

The *in vivo* endocytic capacity of isolated DC subsets was tested by using FITC-conjugated ovalbumin (OVA). OVA-FITC (10 mg/ml) was injected *i.v*. into mice in 300 μl HBSS. Mice were left for 24 hrs before sacrifice and spleen removal. Spleens were dissociated and stained with antibodies.

### CFSE labelling

Cells were labelled with CFSE for analysis of reduction in label as a measure of cell division. Cells were washed and then resuspended in DMEM (100 μl/10^7^ cells). One μl CFSE, final concentration 10 μg/ml, was added and vortexed immediately. Cells were incubated for 5 min. at room temperature with agitation before adding 1 ml sDMEM and washing twice.

### OT-I and OT-II T-cell stimulation assays

Antigen-specific T-cell stimulation capacity of DC was assessed by using CD8^+^ T cells obtained from OT-I mice and CD4^+^ T cells obtained from OT-II mice. These mice express TCR transgenes specific for OVA presented on either MHC-I (OT-I) or MHC-II (OT-II). Hen egg lysozyme (HEL; Sigma-Aldrich, St. Louis, MO, USA) was used as a negative control antigen.

Dendritic cells were pulsed *in vivo* with either 0.5 mg HEL or 0.5 mg OVA administered through *i.v*. injection. After 24 hrs, spleen was collected and the DC subsets stained and sorted as described above. CD4^+^ and CD8^+^ T cells were isolated from LN of OT-II and OT-I TCR-tg mice by magnetic depletion as described above. Sorted cells were incubated with CFSE-labelled T cells (1 × 10^5^ for OT-I, and 1 × 10^4^ for OT-II) in 96-well plates at the following ratios, and collected at 4 days for analysis as for the MLR. DC: T cells for OT-I: 1:10 and 1:100, and for OT-II: 1:1 and 1:5.

## Results

### Identification of cells with CD11c^lo^CD11b^hi^CD8α^−^MHC-II^−^ phenotype in murine spleen, BM and blood

**Figure 1:**
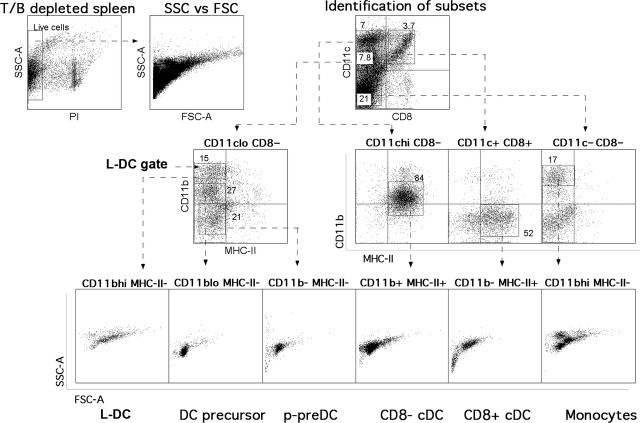
Identification of DC subsets in the spleen. The prevalence of L-DC in relation to other DC subsets was examined by antibody staining of dissociated leucocytes isolated from red blood cell-lysed spleen, BM, LN and thymus. Apart from CD11b and CD11c expression, DC subsets can be further delineated according to CD8α and MHC-II expression. Cells were stained with fluorochrome-labelled antibodies specific for CD11c, CD11b, CD8α and MHC-II. Dead cells were first gated out as PI^+^, followed by analysis of the expression of CD11c *versus* CD8α, and then CD11b *versus* MHC-II. DC subsets were categorized after delineation of CD11c^lo^ and CD11c^hi^ subsets. Cross-hairs were set to exclude background staining on the basis of isotype control antibodies. Side scatter (SSC) *versus* forward scatter (FSC) plots provide an extra parameter for identification of cells based on granularity and size, respectively.

**Figure 2:**
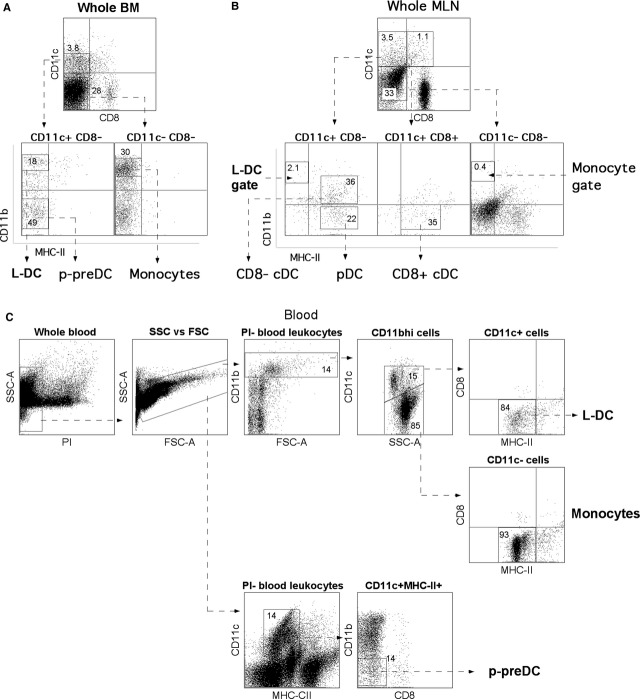
Identification of DC subsets in BM, blood and MLN. The prevalence of L-DC in relation to other DC subsets in BM (A), MLN (B) and blood (C) was examined as described in Figure 1.

As described previously by Tan *et al*. in 2011 11, the *in vivo* counterpart to L-DC represent a distinct subset of CD11c^lo^CD11b^hi^CD8α^−^MHC-II^−^ cells in spleen compared with cDC, pDC and monocytes, and are distinguishable by the selected markers as well as by their characteristically large size. Each of the splenic DC subsets is identified here by using the same gating strategy (Fig. 1). This gating protocol was applied to BM (Fig. 2A), MLN (Fig. 2B) and blood (Fig. 2C) to identify any similar cells. L-DC-like cells, as well as monocytes and p-preDC, were identified in BM and blood but not in MLN.

### L-DC represent a small but significant subpopulation in relation to other DC subsets

Prevalence of L-DC in each organ relative to cDC, pDC and monocytes was analysed by identification of each subset and calculation of its percentage in relation to total leucocyte number in BM or spleen, accounting for per cent depletion of T and B cells in the case of spleen (data not shown): (% cells amongst T-/B-depleted organ)/100 × (% T/B depletion).

Prevalence of each subset amongst total dendritic (CD11c^+^) and myeloid (CD11b^+^) cells was also calculated: (% subset of total organ)/(% dendritic & myeloid cells in organ) × 100.

Means ± SD were calculated for all animals tested (*n* = 7 for spleen, *n* = 6 for BM and MLN, and *n* = 2 for blood) and data are shown in Table 2. L-DC represent the smallest subset of DC in spleen, comprising just 0.07 ± 0.02% of all spleen leucocytes (data not shown) and 5.86 ± 0.30% of all CD11c^+^ and/or CD11b^+^ dendritic/myeloid cells (Table 2). The most abundant subsets in spleen were CD8α^−^ cDC and monocytes, representing equal proportions of the dendritic/myeloid fraction of spleen: 30.20 ± 13.05% for CD8α^−^ cDC and 31.00 ± 13.08% for monocytes. It is not clear whether this variation reflects natural variation between animals or experimental error related to preparation and staining. CD8α^+^ cDC and p-preDC reflected similar proportions (∼17%) of all dendritic/myeloid cells.

**Table 2:** Prevalence (%) of DC subsets and monocytes amongst myeloid and dendritic cell subsets (±SD)

	Spleen			BM			LN			Blood
	+/+	*san/san*	RAG-1−/−	+/+	*san/san*	RAG-1−/−	+/+ (MLN)	*san/san* (CLN)	RAG-1−/− (MLN)	+/+
CD8− cDC	30 ± 13	22 ± 5	2 ± 1	-	-	-	37 ± 7	86 ± 1	99 ± 0	-
CD8+ cDC	17 ± 5	22 ± 3	4.3 ± 1.3	-	-	-	9 ± 2	11 ± 0	1 ± 0	-
L-DC	6 ± 0.3	12 ± 3	-	7 ± 1	-	1.2 ± 0	-	3 ± 1	-	5 ± 2
pDC	–	38 ± 1	14.5 ± 2.7	–	–	6.8 ± 1.6	54 ± 7	–	–	12 ± 1
p-preDC	16 ± 2	–	–	13 ± 5	1 ± 1	–	–	–	–	
Monocytes	31 ± 13	6 ± 5	–	80 ± 5	99 ± 1	92 ± 1.5	–	–	–	82 ± 1
DC prec	–	–	64 ± 1	–	–	–	–	–	–	–
Myeloid prec	–	–	15.3 ± 3.3	–	–	–	–	–	–	–

In BM, cells resembling L-DC represent the smallest subset amongst identified dendritic/myeloid cells with an average representation of 7 ± 1.5%. Plasmacytoid-preDC represent a very variable population, making up 12.67 ± 5% of dendritic/myeloid cells. Again, this could reflect variation between animals or between experiments. Monocytes were by far the largest subset in BM, representing 80.17 ± 5% of all dendritic/myeloid cells. No L-DC were detected in MLN. The DC subsets detected in blood were L-DC (5 ± 2%), p-preDC (12 ± 1%) and monocytes (82 ± 1%) in a composition similar to that observed in BM.

### L-DC have a highly endocytic phenotype

**Figure 3:**
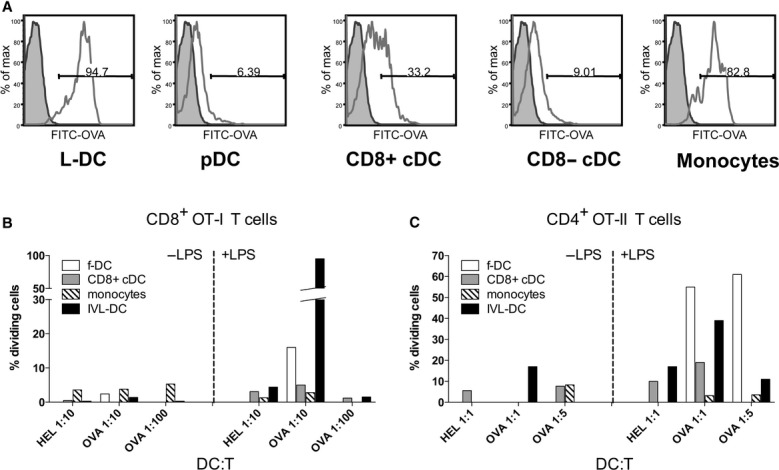
Endocytic and stimulatory capacity of splenic DC subsets. (A) The endocytic capacity of splenic DC subsets was tested through *i.v*. delivery of FITC-conjugated OVA 24 hrs before the animals were killed. Spleens were then collected and stained for DC markers as described previously. Percentages of cells taking up were OVA determined, with gating based on animals not receiving FITC-OVA. (B and C) The ability of splenic DC subsets to take up antigen and present to T cells was investigated by *i.v*. administration of OVA or hen egg lysozyme (HEL; negative control) as in (A) prior to isolation of DC subsets by fluorescence-activated cell sorting. CD4^+^ or CD8α^+^ T cells were isolated from OT-II or OT-I mice, respectively, through magnetic bead depletion and labelled with CFSE. Antigen-presenting cells were then co-cultured with T cells for 4 days in the presence or absence of LPS. (B) Expansion of CD4^+^ T cells, and (C) expansion of CD8α^+^ T cells. Freshly isolated and *in vitro*-stimulated CD11c^+^ DCs (f-DC) were used as a positive control for the assay.

It has already been established that, compared to cDC and monocytes, L-DC are highly endocytic when pulsed with antigen *in vitro*, and are capable of activating CD8^+^ T cells in a mixed lymphocyte reaction 11. To further investigate their functional capacity in terms of their ability to take up antigen *in vivo*, mice received 3 mg FITC-conjugated OVA *i.v*. Mice were killed 24 hrs later and spleens stained for DC subsets to determine OVA-FITC uptake. Figure 3A shows highest level of uptake by monocytes and L-DC (∼90% of cells). Uptake by cDC was comparatively low (9% and 30% for CD8α^+^ cDC and CD8α^−^ cDC, respectively).

In an attempt to distinguish L-DC from monocytes based on function, DC subsets were sorted from animals that had been pulsed with OVA antigen *in vivo* as described above. The sorted cells were then incubated with CFSE-stained CD4^+^ or CD8^+^ T cells from mice expressing OVA-specific TCR (OT-II or OT-I mice), respectively. APCs and T cells were co-cultured with or without 3 μg lipopolysaccharide (LPS) for 4 days before analysis of cell proliferation by flow cytometry. The T-cell proliferative response was therefore related to *in vivo* uptake capacity for antigen by each subset.

As shown in Figure 3B, the strongest effect on CD8^+^ OT-I T-cell proliferation was induced by L-DC, but only after culture in the presence of LPS. This suggests that L-DC are very capable activators of OT-I T cells, and are both accessible to blood-borne antigen and capable of high uptake compared with other subsets. In particular, whilst monocytes and L-DC have very similar marker expression (Table 1), monocytes had no capacity to stimulate either CD4^+^ or CD8^+^ T cells.

None of the isolated subsets showed particularly high capacity to activate OT-II CD4^+^ T cells. T-cell proliferation was weak, but OVA-specific. A response was seen for freshly isolated CD11c^+^ splenic DC and for L-DC, although the latter response was not improved by addition of LPS (Fig. 3C).

### Prevalence of L-DC in sanroque animals

To investigate possible sources of a higher yield of L-DC, we investigated their prevalence in mutant mouse strains. As L-DC are thought to derive from the myeloid lineage, two available mutant mouse strains with a known increase in myelopoiesis were investigated: s*anroque* 12 and *Rag*^*−/−*^. Both strains are on the same C57BL/6J genetic background as the wild-type (+/+) mice analysed above so that any perturbation in L-DC number in mutant strain would therefore be due entirely to the mutation.

*Sanroque* mice were developed by Prof. Chris Goodnow and Dr. Carola Vinuesa (JCSMR: ANU), and lack the ubiquitin ligase Roquin 12. Mice homozygous for the mutation are labelled *san/san*. These mice have severe autoimmune disease, and all develop systemic lupus erythematosus. Mice are in a perpetual state of inflammation because of autoimmunity 13 and appear to have a higher prevalence of myeloid cells 12. Consequently, it was thought that the *sanroque* mouse model may show an enriched population of L-DC, with or without other myeloid lineage cells.

**Figure 4:**
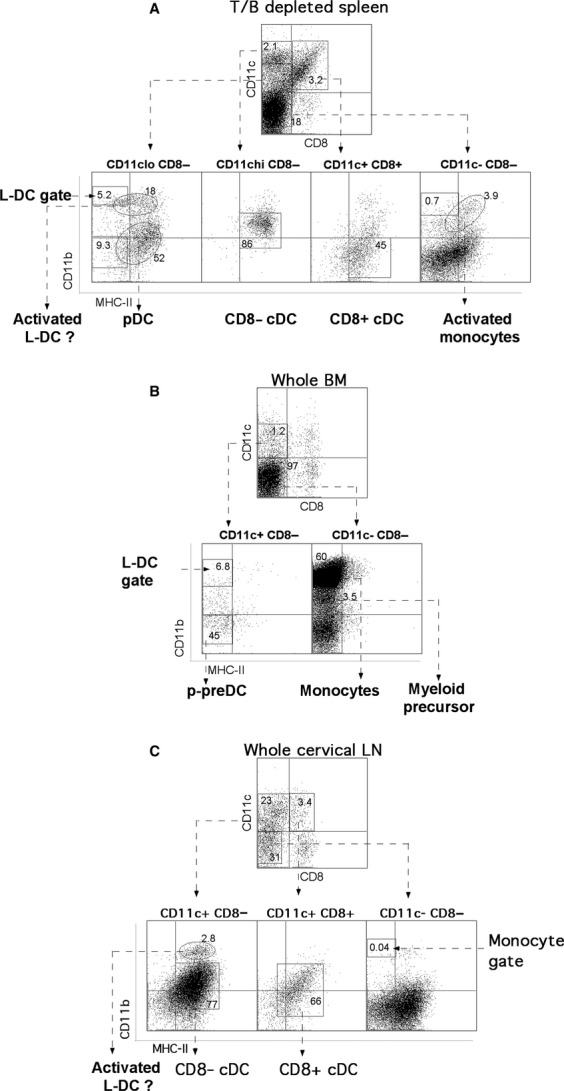
Distribution of dendritic/myeloid subsets in *sanroque* mice. Spleen, BM and LN cells from two 6-month-old female *sanroque* (*san/san*; C57BL/6J) mice were prepared and stained with antibody as described previously in the legend to Figure 1. Representative analysis of one mouse is shown. Cross-hairs delineated background binding because of isotype control antibodies and identifiable subsets of cells. Gates were set based on antibody binding to a representative age-matched control (+/+) C57BL/6J mouse, and were transferred to plots for the mutant animals to compare relative location of subsets. Rectangular gates represent those transferred from the +/+ control, while circular gates identify new populations detected in mutant mice. For the spleen, T-/B-depleted cells were stained with antibodies specific for CD11c, CD11b, CD8α and MHC-CII. Live cells were gated by PI staining (data not shown) and CD11c^lo^CD8α^−^, CD11c^hi^CD8α^−^, CD11c^hi^CD8α^+^ and CD11c^−^CD8α^−^ subsets gated for delineation of CD11b and MHC-II staining. This allowed delineation of L-DC, pDC, CD8α^+^ cDC, CD8α^−^ cDC and monocytes. Analysis of *sanroque* spleen (A), BM (B) and cervical LN (C) are shown.

The presence of L-DC in mutants was checked by staining for DC markers as for +/+ mice. Flow cytometry gates from the +/+ control were overlaid on to those for mutant mice. Circular gates, shown in Figure 4, were then used to denote populations present in mutant, but not +/+ animals. Spleen (A), BM (B), and mesenteric (data not shown) and cervical LN (CLN) (C) were prepared for analysis. Cervical lymph nodes were taken from these animals because of their obvious enlargement.

Figure 4 shows how the general profile of DC subsets in spleen, BM and CLN of *san/san* mice differs from that of +/+ mice. The CD11b^hi^MHC-II^−^ population gated from the +/+ CD11c^lo^CD8α^−^ cell subset has been classified as L-DC, and this population shows characteristic high FSC (data not shown). However, it appears that any L-DC have gained MHC-II expression in *san/san* mice, a phenotype that is possible because of the constitutive inflammatory state of *san/san* animals. Several other cell populations in *san/san* spleen also appear to have altered phenotypes, such as monocytes, which have gained MHC-II expression. Furthermore, pDC derived from the CD11c^lo^CD8α^−^ gate have gained MHC-II, and perhaps low CD11b expression, which could represent activated pDC.

L-DC are not present in BM of *san/san* mice in any significant number, such that their existence is debatable. As observed in +/+ mice, there is no detectable population of pDC; however, *san/san* animals appear to have a significant population of p-preDC (Fig. 4B). There is also increased representation of a CD11c^−^CD11b^lo^CD8α^−^MHC-II^−^ subset (Fig. 4B) that may represent an expanded population of myeloid precursors. In *san/san* BM, monocytes dominate in cell number, representing roughly double the percentage present in +/+ animals (Fig. 4B and Table 2). This reflects the previously observed increase in cells of the myeloid lineage in *san/san* mice 12.

Dendritic cells subsets in MLN (data not shown) and CLN (Fig. 4C) of *san/san* mice differ noticeably from those in +/+ mice. A population of CD11c^lo^CD11b^hi^CD8α^−^MHC-II^lo^ cells is present, which reflect activated myeloid DC in phenotype, and possibly activated L-DC, although their size on FSC is not as large as L-DC detected in +/+ spleen and BM, and cells also express MHC-II.

Table 2 contains a summary of the percentage distribution of each identifiable dendritic and myeloid cell subset amongst total spleen, BM and CLN dendritic/myeloid cells. These data are based on mean ± SD (*n* = 2). In the spleen, percentages of all DC subsets are reduced by about half in *san/san* mice compared with +/+ animals, with the exception of CD8α^+^ cDC, whose percentages remain similar, although not statistically significant (*P* ≤ 0.05). The drop in monocytes observed in the spleen could reflect inflammation-induced migration to blood, where levels of myeloid-derived cells are uncharacteristically high.

### L-DC are absent in Rag^−/−^ mice

**Figure 5:**
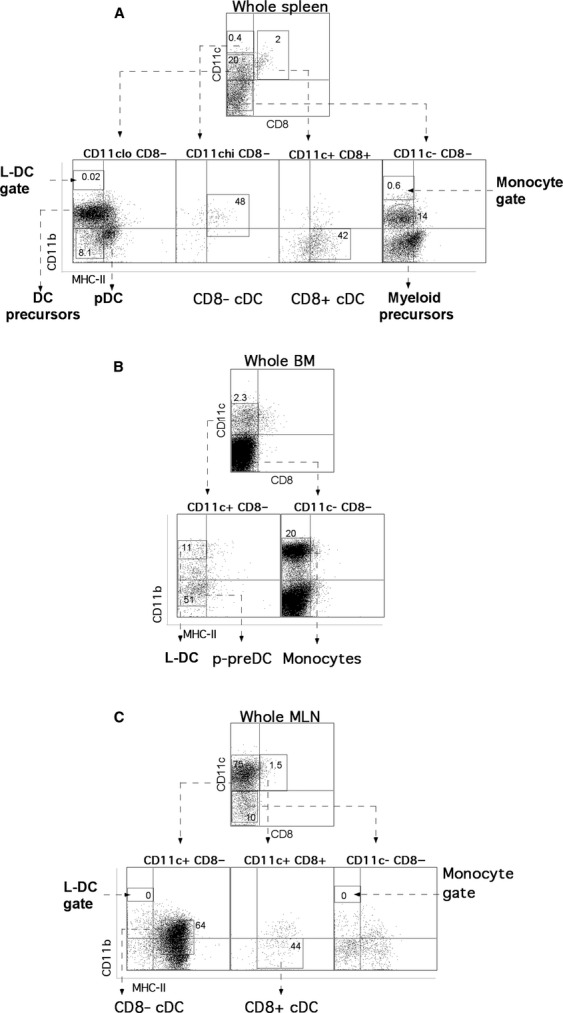
Distribution of DC subsets in *Rag*^*−/−*^ mice. Twelve week-old Rag^*−/−*^ mice were obtained from the Animal Services Division (JCSMR). Spleen, bone marrow (BM), LN and thymus were isolated and cells prepared and stained as described in the legend to Figure 1. A 12-week-old female C57BL/6J mouse was used as a +/+ control. Cross-hairs were set based on binding of isotype control antibodies and identifiable subsets of cells. Gates were set on the control and transferred to the plots of cells from two *Rag*^*−/−*^ mice, with rectangular gates representing those transferred from the +/+ control, and circular gates identifying populations not present on the control plots. Live cells were gated using PI staining (data not shown), and CD11c^lo^CD8α^−^, CD11c^hi^CD8α^−^, CD11c^hi^CD8α^+^ and CD11c^−^CD8α^−^ populations were gated for delineation of CD11b and MHC-II expression on spleen (A), BM (B) and MLN (C) of two *Rag*^*−/−*^ mice.

*Rag*^*−/−*^ mice lack the gene encoding recombinases essential for rearrangement of TCR and immunoglobulin genes in T and B cells. In the absence of T- and B-cell development, these mice have increased myelopoiesis. Monocytes, cDC and pDC are all much harder to identify as clear subsets in spleen of *Rag*^*−/−*^ compared with +/+ animals. The majority of cells are smaller in size and reflect myeloid or DC precursors. No population of L-DC was detectable in the spleen (Fig. 5A). Major populations of CD11c^lo^CD11b^lo^ and CD11c^−^CD11b^lo^ cells reflect small undifferentiated cells. This expanded population is consistent with less mature myeloid cell development because of the absence of T cell-derived colony-stimulating factors and cytokines. L-DC are detectable in *Rag*^*−/−*^ BM in numbers less than in +/+ (Fig. 5B and Table 2). Monocyte and p-preDC populations are also detected. As with the spleen, there is an increased number of CD11c^−^ cells, reflecting undifferentiated cells. The layout of cell populations in BM from *Rag*^*−/−*^ mice was very similar to that in +/+ mice, suggesting no significant effect of the mutation on BM cell populations apart from the reduced number of L-DC (Fig. 5B). This contrasts with spleen DC subset development, which must be dependent on T/B-derived haematopoietic factors. The populations present in *Rag*^*−/−*^ MLN appear to be similar to those present in +/+ mice, except that the cDC populations have higher levels of CD11b expression (Fig. 5C). Relative percentages of each subset are summarized in Table 2. This shows that *Rag*^*−/−*^ spleen contains only cDC and pDC, and no L-DC or monocytes, with the per cent of CD8α^−^ and CD8α^+^ cDC dramatically reduced, as are L-DC percentages in BM.

## Discussion

This paper aims to further characterize L-DC on the basis of presence in lymphoid organs other than the spleen and the relative size of the cell population. In keeping with these aims, populations of cells matching L-DC marker expression were clearly present as distinct populations in spleen, blood and also BM, representing ∼6, ∼5 and ∼7% of all DC and monocytes in the spleen, blood and BM, respectively. Study of the steady-state function of L-DC is necessary to further characterize this unique subset. Moreover, the relationship between the populations identified in spleen and BM needs to be established. Based on evidence from long-term cultures, L-DC are spleen-derived cells, so it is important to establish the origin of the population identified in BM in terms of the site of development and the source of the precursor. Previous work in the laboratory has shown that *in vitro* culture of BM cells, probably containing HSC, over permissive spleen-derived stroma gives rise to L-DC 14, reflecting the hypothesis that L-DC derive from HSC and are directed towards L-DC by the spleen stroma 15. It remains to be seen if BM contains a similar L-DC-directing supportive cell line. Preliminary functional studies performed on both spleen- and BM-derived L-DC show that BM L-DC may have less capacity to activate T cells compared with spleen-derived L-DC.

In this paper, we investigated the presence of L-DC in lymphoid organs. It is also possible that these cells are present in tissue sites, such as the gut or the skin. Given the lack of L-DC in mesenteric lymph nodes, however, which drain the gut, it is unlikely that this subset is present at this site. Furthermore, previous studies have shown that L-DC lack langerin expression and are therefore not likely to be skin-related. The fact that L-DC are suggested to develop in the spleen from HSC in a specialized niche 15 suggests that the cells have a specialised role within this organ. Study of L-DC prevalence in other organs, however, is a possible direction of future investigations.

It is clear that L-DC represent a cell population distinct from cDC and pDC due particularly to their characteristically low CD11c and high CD11b expression. High levels of CD11b on the cell surface suggest that the cells are of myeloid origin. Their presence in the spleen could indicate a role in screening blood for either pathogen-related antigen or self-antigen. Indeed, their accessibility to blood-borne antigen is noted here to be far superior to other DC subsets. Whilst CD8α^+^ cDC and fresh splenic CD11c^+^ DC are known to be very good activators of both CD8^+^ and CD4^+^ T cells, neither could take up sufficient blood-borne antigen to induce proliferation of TCR-tg T cells in the experiment described here involving *in vivo* priming. In contrast, L-DC show a very strong T-cell activation response because of high accessibility to antigen as well as strong cross-presenting capacity in the presence of LPS. The latter is a potent immunomodulatory molecule, signalling through toll-like receptor (TLR) 4 on some APC 16. It is also likely that L-DC respond to activation through up-regulation of costimulatory molecules such as CD80, and production of cytokines, which are so far not defined for this subset 17. Further studies involving *in vitro* activation of carefully isolated subsets by cell sorting will be needed to determine which cytokines are produced by L-DC in comparison with other DC subsets. Studies are still in progress to define a mutant mouse strain or a protocol, which gives numbers of cells suitable for more definitive studies of L-DC phenotype and function.

The exact location of L-DC in the spleen and how this location changes in the event of inflammation are still under investigation. This knowledge will almost certainly assist in determination of putative functions for L-DC. The lack of MHC-II surface expression on L-DC in the steady state is significant and could represent a tolerogenic role for these cells in the absence of inflammation. Alternatively, low MHC-II expression and high MHC-I expression 11 may indicate a CD8^+^ T cell-specific role for L-DC, for example through cross-presentation.

A cell phenotype of CD11c^lo^CD11b^hi^CD8α^−^MHC-II^−^ could also be claimed to fit the phenotype of activated monocytes. Further analysis of size and marker expression, however, rules against this possibility, as it has been shown that L-DC express higher levels of MHC-I than monocytes and are able to activate CD8 T cells in our assays (11 and Fig. 3B). Future studies will aim to determine markers and functions that serve to distinguish these cell types.

Given the predicted myeloid origin of L-DC indicated by the high level of CD11b expression, the existence of L-DC was examined in mutant mouse strains known to have perturbations in myelopoiesis. Mice lacking certain genes are a useful way to study cell development for two reasons. First, mutants could be a better source of cells either because of overproduction of the cell type in question or absence of other cell types. Secondly, a mutant mouse strain may be of interest if it specifically lacks the cell type of interest in terms of identifying developmental origins. The phenotypes of L-DC in the mutant mouse models *sanroque* and *Rag*^*−/−*^ reflect the state expected because of the animal models. For example, L-DC in *san/san* animals show an up-regulation of CD8α and MHC-II expression, possibly reflecting activation of cells because of the constant inflammatory state induced by autoimmunity. In *Rag*^*−/−*^ animals, by comparison, L-DC are not evident in the spleen, possibly reflecting an inability to develop in the absence of T- or B-cell-related factors. Crowley *et al*. 18 showed that although lymphocytes are generally not essential to the organization of the spleen and the presence of most DC subsets, lack of lymphocytes had a minor effect on marginal zone myeloid DC survival and maturation, which, they suggested, could be due to a lack of B cell-derived factors such as macrophage inflammatory factors α and β. This was reflected in our experiments, where we observed cDC populations in *Rag*-deficient mice, but report a lack of L-DC, which are thought to be of a myeloid origin. As we hypothesize that L-DC develop in the spleen 15, it is likely that similar factors are necessary for L-DC development from spleen-resident HSC or progenitors. In both cases, the mutant strain of interest did not serve to provide higher numbers of L-DC required to perform a more complete analysis of their function. These experiments have demonstrated that the same L-DC are not present in the mutant strains investigated. The distribution and phenotypes of the DC subsets and monocytes identified, however, are indicative of the known characteristics of each mutant. For example, L-DC in the spleen of *san/san* animals could have an inflammatory phenotype, whilst more undifferentiated myeloid cells are present in spleen of *Rag*^*−/−*^ animals.

## Conclusion

From these experiments, it is evident that L-DC represent a small but distinct subset of dendritic-like cells in the spleen. A similar cell type is also present in BM, although future studies will need to determine whether they represent the same subset. Although the function of L-DC is still under investigation, it is thought that they reflect a unique subset that will demonstrate unique function specific to their predominant location in spleen and blood.
